# Lactoferrin Efficiently Counteracts the Inflammation-Induced Changes of the Iron Homeostasis System in Macrophages

**DOI:** 10.3389/fimmu.2017.00705

**Published:** 2017-06-15

**Authors:** Antimo Cutone, Luigi Rosa, Maria Stefania Lepanto, Mellani Jinnett Scotti, Francesca Berlutti, Maria Carmela Bonaccorsi di Patti, Giovanni Musci, Piera Valenti

**Affiliations:** ^1^Department of Public Health and Infectious Diseases, Sapienza University, Rome, Italy; ^2^Department of Biochemical Sciences, Sapienza University, RomeItaly; ^3^Department of Biosciences and Territory, University of Molise, Pesche, Italy

**Keywords:** lactoferrin, iron, homeostasis, cytokines, inflammation, ferroportin, ceruloplasmin, macrophages

## Abstract

Human lactoferrin (hLf), an 80-kDa multifunctional iron-binding cationic glycoprotein, is constitutively secreted by exocrine glands and by neutrophils during inflammation. hLf is recognized as a key element in the host immune defense system. The *in vitro* and *in vivo* experiments are carried out with bovine Lf (bLf), which shares high sequence homology and identical functions with hLf, including anti-inflammatory activity. Here, in “pure” M1 human macrophages, obtained by stimulation with a mixture of 10 pg/ml LPS and 20 ng/ml IFN-γ, as well as in a more heterogeneous macrophage population, challenged with high-dose of LPS (1 µg/ml), the effect of bLf on the expression of the main proteins involved in iron and inflammatory homeostasis, namely ferroportin (Fpn), membrane-bound ceruloplasmin (Cp), cytosolic ferritin (Ftn), transferrin receptor 1, and cytokines has been investigated. The increase of IL-6 and IL-1β cytokines, following the inflammatory treatments, is associated with both upregulation of cytosolic Ftn and downregulation of Fpn, membrane-bound Cp, and transferrin receptor 1. All these changes take part into intracellular iron overload, a very unsafe condition leading *in vivo* to higher host susceptibility to infections as well as iron deficiency in the blood and anemia of inflammation. It is, therefore, of utmost importance to counteract the persistence of the inflammatory status to rebalance iron levels between tissues/secretions and blood. Moreover, levels of the antiinflammatory cytokine IL-10 were increased in cells treated with high doses of LPS. Conversely, IL-10 decreased when the LPS/IFN-γ mix was used, suggesting that only the inflammation triggered by LPS high doses can switch on an anti-inflammatory response in our macrophagic model. Here, we demonstrate that bLf, when included in the culture medium, significantly reduced IL-6 and IL-1β production and efficiently prevented the changes of Fpn, membrane-bound Cp, cytosolic Ftn, and transferrin receptor 1 in “pure” M1 macrophages, as well as in the more heterogeneous macrophage population. In addition, the decrease of IL-10 induced by the LPS/IFN-γ mix was counteracted by bovine lactoferrin. Several drugs capable of modulating macrophagic phenotypes are emerging as attractive molecules for treating inflammation, and in this sense, bovine lactoferrin is no exception.

## Introduction

Iron is essential for living organisms, being required in many proteins for a broad range of vital functions, such as oxygen transport and energy production. However, it can be toxic when in excess due to its ability to partially reduce oxygen, thus generating reactive oxygen species, which are known to cause tissue injury and organ failure by damaging a number of cellular components, including DNA, proteins, and membrane lipids. This has led to the evolution of strictly controlled pathways of iron uptake and release to minimize its deficiency or excess (1).

Systemic iron homeostasis is tightly controlled in humans. Both iron acquisition by enterocytes and iron recycling by macrophages are regulated through iron absorption, storage, and export, as mammals lack a direct iron excretion system (2, 3). In physiological conditions, enterocytes absorb 1–2 mg/day of iron, while macrophages recycle about 20 mg/day of iron from senescent erythrocytes. Consequently, recycled iron is the larger source of iron in the plasma compared to that exported by enterocytes and hepatocytes.

Dietary iron enters the body at the duodenal level through the concerted action of the ferrireductase DCYTB and the metal importer DMT-1 (4). Enterocytes then pour iron in the plasma through ferroportin (Fpn), the only iron exporter identified in mammalian cells, situated on the basolateral membrane of the enterocytes but also present in all other cell types involved in iron export, including hepatocytes, macrophages, and placental syncytiotrophoblasts (5, 6). Another controller of iron homeostasis is hepcidin, a peptide hormone synthesized by hepatocytes (7) that induces the degradation of Fpn, thus hindering iron export and causing intracellular iron overload (1). On the other hand, a positive regulation of Fpn is exerted by a companion ferroxidase that not only has been shown to stabilize Fpn on the plasma membrane (8) but is also pivotal to convert the exported ferrous iron into its ferric form to allow its binding to serum transferrin (9). In mammals, two homologous ferroxidases exert this role: hephestin, mainly expressed by small intestine, spleen and kidney cells, and ceruloplasmin (Cp), which is synthesized by hepatocytes, macrophages, and immune cells (9). In turn, two isoforms of Cp are found: soluble Cp is mainly found in plasma, while the glycosyl-phosphatidyl-inositol-anchored form (Cp-GPI) has been identified in numerous tissues in addition to macrophages and immune cells (9). Once in the portal circulation, ferric iron chelated by transferrin is transported to sites of use and storage and released through receptor-mediated endocytosis (10). Uptaken iron is either promptly utilized by the cell or stored into cytosolic ferritin (Ftn), a protein composed of 24 subunits endowed with ferroxidase activity, able to harbor up to 4,500 iron atoms per molecule as oxy-hydroxide micelles (11).

During infection and inflammation, iron homeostasis is grossly perturbed, leading to iron disorders. Enterocytes and macrophages become iron overloaded, thus increasing the host susceptibility to infections (12). On the other hand, the intracellular iron overload is associated to the decrease of Fpn synthesis, leading to diminished transport of iron into plasma with consequent iron deficiency anemia (IDA) and anemia of inflammation (AI) (12, 13). The cytokine-induced “anemia of inflammation” has been primarily considered as a defense mechanism to limit iron availability to pathogens in the blood (14, 15). However, in light of the ability of bacteria to enter and survive inside macrophages, this concept should be critically reconsidered. In fact, intracellular iron retention could be an inducer of intracellular pathogens’ growth and lead to increased infection severity. It is, therefore, of utmost importance to counteract the persistence of the inflammatory status to rebalance iron levels between tissues/secretions and blood.

In this respect, specific cells express molecules that sequester iron to promote host defense. In particular, many mucosal epithelia secrete human lactoferrin (hLf), an 80-kDa iron-binding cationic glycoprotein, which, at variance with transferrin, is able to chelate two Fe (III) per molecule with high affinity even at the very low pH values, characteristic of inflamed and infected sites (16). It is also secreted and released by neutrophils during inflammation. hLf is recognized as a key element in the host immune defense system (17).

In addition to the antimicrobial properties dependent and independent from its iron-binding ability, hLf exhibits a variety of effects on the host immune system, ranging from inhibition of inflammation to promotion of both innate and adaptive immune responses (17, 18).

Bovine Lf (bLf), which shares high sequence homology with the human protein, is also a multifunctional glycoprotein with identical antibacterial, antifungal, antiviral, antiparasitic (18), anti-inflammatory, and immunomodulatory activities of hLf (19, 20). The majority of the *in vitro* studies as well as clinical trials have been carried out using bLf, generally recognized as a safe substance by Food and Drug Administration (USA) and available in large quantities.

Evidence indicates that bLf modulates inflammation by affecting expression of cytokines, chemokines, and other effector molecules. For instance, oral administration of bLf modulates the expression of IL-6, the main cytokine involved in inflammatory and iron homeostasis, reverting homeostasis disorders in pregnant women suffering from IDA and AI (21–23).

Previously, we demonstrated that bLf affects iron homeostasis in inflamed models of both epithelial and macrophagic cells by inhibiting IL-6 production and rescuing the expression of the iron exporter Fpn (24, 25).

Although the mechanisms underlying bLf anti-inflammatory properties have not been fully elucidated yet, its interaction with macrophages may play a critical role.

In this work, we have extended our study, investigating the effect of bLf on the expression of all pivotal proteins (Fpn, membrane-bound Cp, cytosolic Ftn, transferrin receptor 1, and cytokines) involved in mammalian iron and inflammatory homeostasis in a model of inflamed human macrophagic THP-1 cells, challenged with a mixture of LPS and IFN-γ or with LPS alone. The rationale is that macrophages can adapt their phenotype in response to different environmental stimuli and that the iron system proteins are expressed at different extent in different macrophagic phenotypes. Here, we reported the effect of bLf added both to a classical mixture of low concentrations of LPS and IFN-γ, known to induce a pure M1 polarization, and to high concentrations of LPS, known to induce a mixed inflammatory M1/tolerogenic M2 phenotypic population on the expression of iron and inflammatory homeostasis.

## Materials and Methods

### Lactoferrin

Highly purified bLf was kindly provided by Morinaga Milk Industries Co., Ltd. (Tokyo, Japan). The purity of bLf was checked by SDS-PAGE and silver nitrate staining, while its concentration was assessed by UV spectroscopy on the basis of an extinction coefficient of 15.1 (280 nm, 1% solution). The bLf iron saturation was about 20% as detected by optical spectroscopy at 468 nm on the basis of an extinction coefficient of 0.54 (100% iron saturation, 1% solution). LPS contamination of bLf, estimated by Limulus Amebocyte assay (Pyrochrome kit, PBI International), was equal to 0.7 ± 0.06 ng/mg of bLf. Before biological assays, bLf solution was sterilized by filtration using 0.2 µm Millex HV at low protein retention (Millipore Corp., Bedford, Mass.). In all experiments, bLf was used at a non-cytotoxic concentration corresponding to 100 µg/ml.

### Cell Line and Culture Condition

THP-1 cells, a myelomonocytic cell line derived from the blood of a 1-year-old boy with acute monocytic leukemia (ECACC, European Collection of Cell Cultures), were maintained in RPMI 1640 medium (Euroclone, Italy), supplemented with 10% fetal calf serum and 2 mM glutamine, in an atmosphere of 95% air and 5% CO_2_. Cells, which grow spontaneously in loose suspension under these conditions, were subcultured twice a week by gentle shaking followed by pelleting and reseeding at a density of approximately 10^6^ cells/ml.

### Stimulation of Differentiated THP-1 with LPS and IFN-γ

THP-1 cells were differentiated in macrophages by incubation in 25 cm^2^ flasks at a density of approximately 10^6^ cells/ml in 5 ml of RPMI (supplemented with 100 µM penicillin–streptomycin, 2 mM glutamine, 10% fetal calf serum) containing 0.16 µM phorbol myristate acetate (PMA, Sigma Chemical Co.) for 48 h, at 37°C in an atmosphere of 95% air and 5% CO_2_ (26). Differentiated THP-1 cells were washed twice with 5 ml of PBS without calcium and magnesium and treated with 10 pg/ml LPS from *E. coli* (InvivoGen, USA) and 20 ng/ml IFN-γ (Sigma, Italy), or with 1 µg/ml LPS. The stimulations were carried out for 48 h in an atmosphere of 95% air and 5% CO_2_, at 37°C. 100 µg/ml bLf were added to adherent THP-1 cells, without removing the culture medium, after 3 and 24 h from the stimulation. After 48 h of incubation, the supernatants were harvested, aliquoted, and stored at −80°C for cytokines quantitation. The adherent cells were scraped in 2 ml of PBS containing 1 mM phenylmethylsulfonyl fluoride (PMSF), pelleted by centrifugation at 2,500 × *g* for 5 min, and stored at −80°C for protein analysis.

### Cytokine Analysis

Quantitation of IL-6, IL-1β, and IL-10 was performed on cell monolayer supernatants by ELISA, using Human ELISA Max Deluxe Sets (BioLegend, USA).

### Western Blots

THP-1 cells (about 5 × 10^6^ cells) were lysed in 300 µl of lysis buffer (25 mM 3-morpholinopropane-1-sulfonic acid pH 7.4/150 mM NaCl/1% Triton containing 1 mM PMSF, 2 µM leupeptin, and pepstatin) in ice for 1 h. Total protein content of samples was measured by Bradford assay. For SDS-PAGE, 20 µg of total protein in SDS sample buffer containing 1,4-dithiothreitol, were heat-treated (except for Fpn) and loaded. For Western Blot analysis, primary antibodies used were: monoclonal anti-Fpn 31A5, generously provided by T. Arvedson (Amgen) (1:10,000) (27), monoclonal anti-transferrin receptor 1 (anti-TfR) (Santa Cruz, CA, USA) (1:5,000), polyclonal anti-Ftn (Santa Cruz, CA, USA) (1:10,000), monoclonal anti-actin (Santa Cruz, CA, USA) (1:10,000), and polyclonal anti-HCP (Dako, USA) (1:10,000). After incubation with the appropriate secondary Horseradish Peroxidase-conjugated antibody, blots were developed with Enhanced ChemiLuminescence (ECL Prime) (GE Healthcare, UK). Protein levels were normalized on actin by densitometry analysis, performed with ImageJ.

### Statistical Analysis

All experiments were run at least in triplicate. Results are expressed as mean ± SE. Statistical analysis was performed with GraphPad Prism and analysis of variance was used to compare quantitative data populations with normal distribution and equal variance.

## Results

### THP-1 and Inflammation: Aging Effect on IL-6 Production

**Figure 1:**
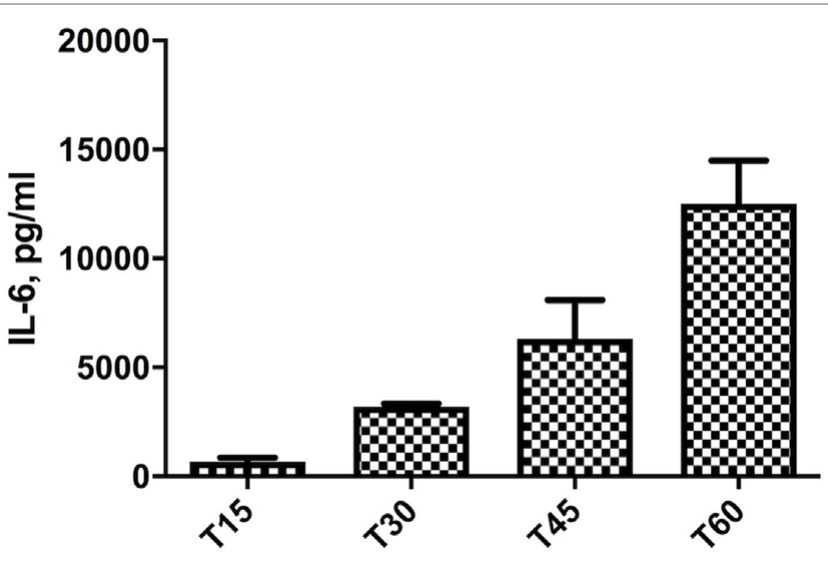
Increase of IL-6 levels in THP-1 cells, differentiated in macrophages by phorbol myristate acetate treatment, cultured for different times. T15–T60 refer to days after the cell batch had been thawed.

Preliminary experiments showed that THP-1 cells are usually very prone to rapid aging. As shown in Figure 1, THP-1 cells, differentiated in macrophages by PMA treatment, show an exponential increase of IL-6 production, starting from the T15, corresponding to day 15 from thawing, to T60. This sort of auto-inflammation process results in non-reproducible response to stimuli during cell aging. For this reason, for all experiments shown in this work, we used cells before T20.

### Bovine Lactoferrin Counteracts the THP-1 Inflammatory Phenotype Induced by LPS and IFN-γ

To induce the inflammatory macrophagic phenotype M1, cells were stimulated with 10 pg/ml LPS and 20 ng/ml IFN-γ, alone or in combination, in the presence or absence of 100 µg/ml bLf.

**Figure 2:**
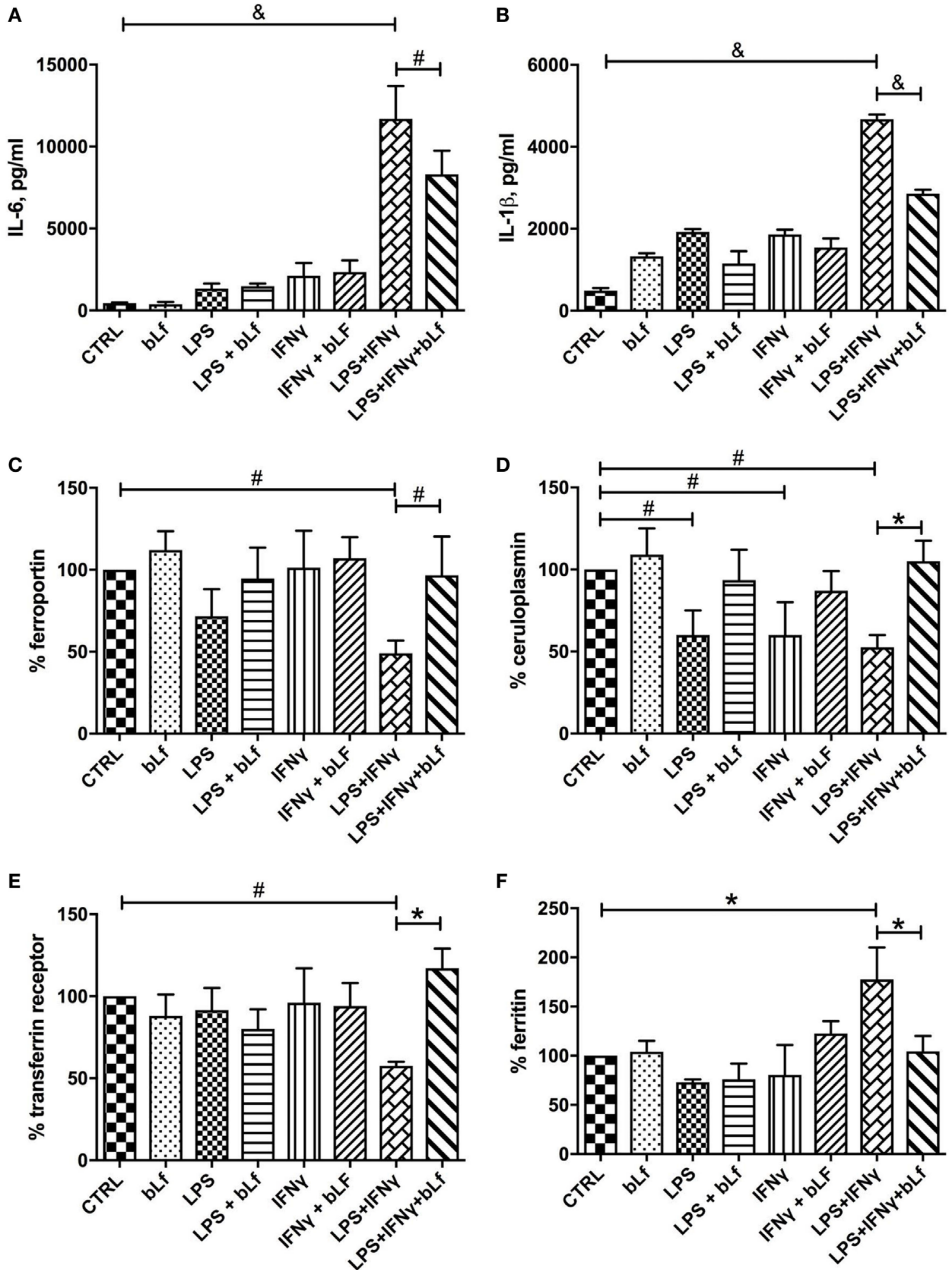
Changes in IL-6 **(A)**, IL-1β **(B)**, ferroportin **(C)**, membrane-bound ceruloplasmin **(D)**, transferrin receptor 1 **(E)**, and cytosolic ferritin **(F)** levels in THP-1 cells stimulated with a mixture of 20 ng/ml IFN-γ and 10 pg/ml LPS, in the presence or absence of 100 μg/ml bLf. ^#^*p* < 0.05; *p < 0.01; ^&^*p* < 0.001.

As shown in Figures 2A,B, levels of both pro-inflammatory cytokines analyzed, IL-6 and IL-1β, significantly increased upon treatment with the mixture, with bLf counteracting the increase by ca. 30 and 40%, respectively. Of note, no significant effects on IL-6 and IL-1β synthesis were observed in samples stimulated with LPS or IFN-γ alone, as well as for bLf on unstimulated cells.

Ferroportin and Cp-GPI synthesis followed the same pattern, with expression of both proteins severely impaired when cells were challenged with the mixture of LPS and IFN-γ (Figures 2C,D). In the case of Fpn, single treatments (i.e., LPS or IFN-γ alone) were less effective in downregulating the protein production, with LPS being more effective than IFN-γ (Figure 2C). On the other hand, single treatments downregulated Cp-GPI synthesis as efficiently as the LPS/IFN-γ mix (Figure 2D). bLf significantly rescued the expression of both proteins in cells stimulated with both LPS and IFN-γ. In the case of Cp-GPI, the recovery was not significant when related to single treatments (*p* values higher than 0.05). The concerted regulation of Fpn and Cp-GPI is consistent with the known functional connection between the two proteins (9).

Expression of TfR1 and Ftn was also found to change, and again production of the relative proteins was significantly affected only by the combined treatment with both LPS and IFN-γ, while being essentially unchanged after single stimulatory treatments. However, TfR1 expression was ca. 40% downregulated by the mixture (Figure 2E), while Ftn production increased about twofold upon stimulation with LPS and IFN-γ (Figure 2F). In any case, the presence of bLf totally prevented the effects of the pro-inflammatory mixture.

**Figure 3:**
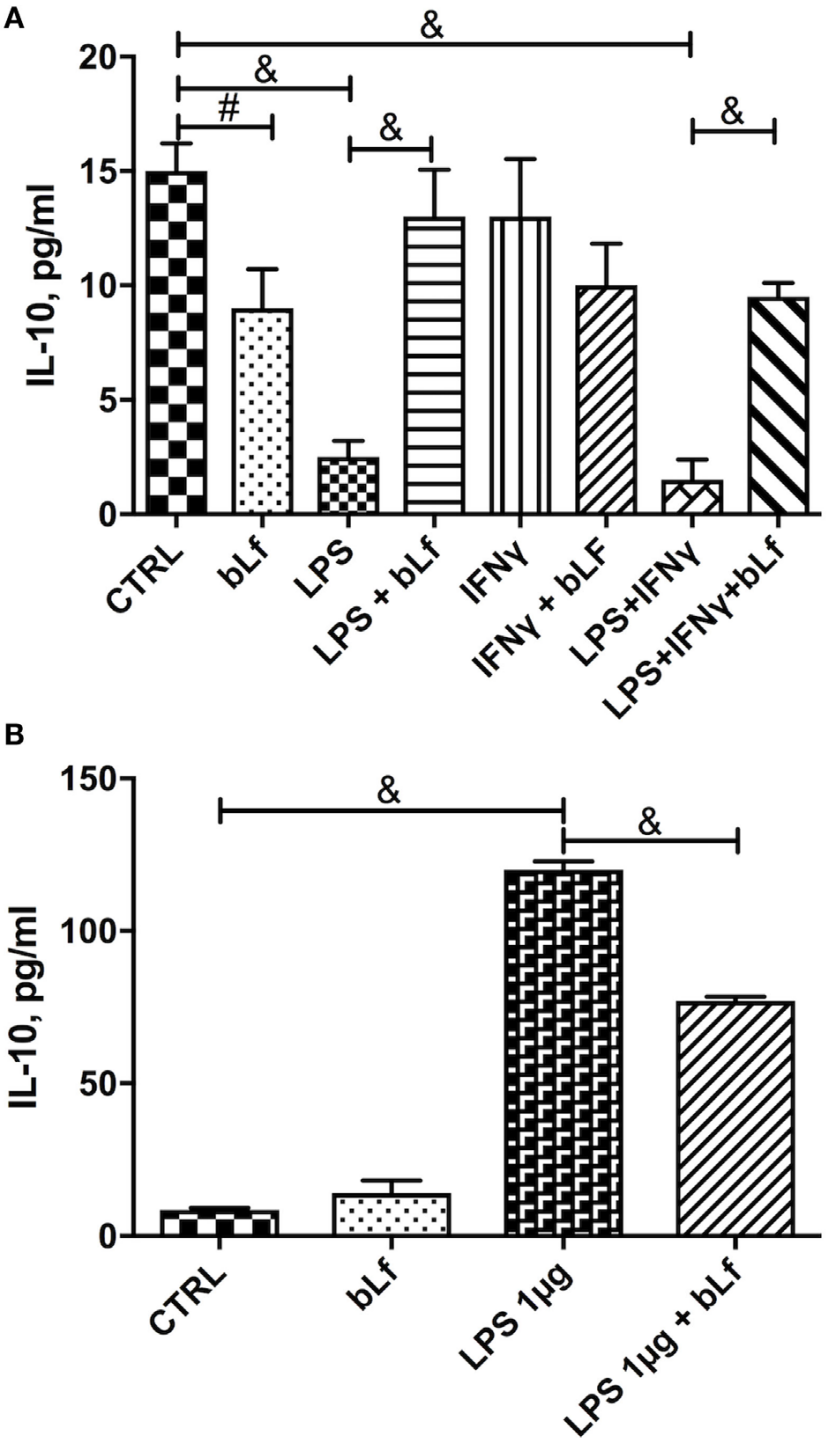
Changes in IL-10 levels in THP-1 cells stimulated with **(A)** a mixture of 20 ng/ml IFN-γ and 10 pg/ml LPS or **(B)** LPS 1 µg/ml in the presence or absence of 100 µg/ml bovine Lf (bLf) (^#^*p* < 0.05; *p < 0.01; ^&^*p* < 0.001).

When IL-10 was measured as a marker of the tolerogenic, anti-inflammatory status of macrophages, it turned out that the treatment with the LPS/IFN-γ mixture dramatically reduced the cytokine levels (Figure 3A). Again, when cells were stimulated in the presence of bLf, this IL-10 decrease was almost completely abolished, suggesting that bLf effectively acts as an anti-inflammatory molecule under these conditions.

### Bovine Lactoferrin Reduces Inflammation and Rebalances Iron Homeostasis in THP-1 Cells Challenged with 1 µg/ml LPS

**Figure 4:**
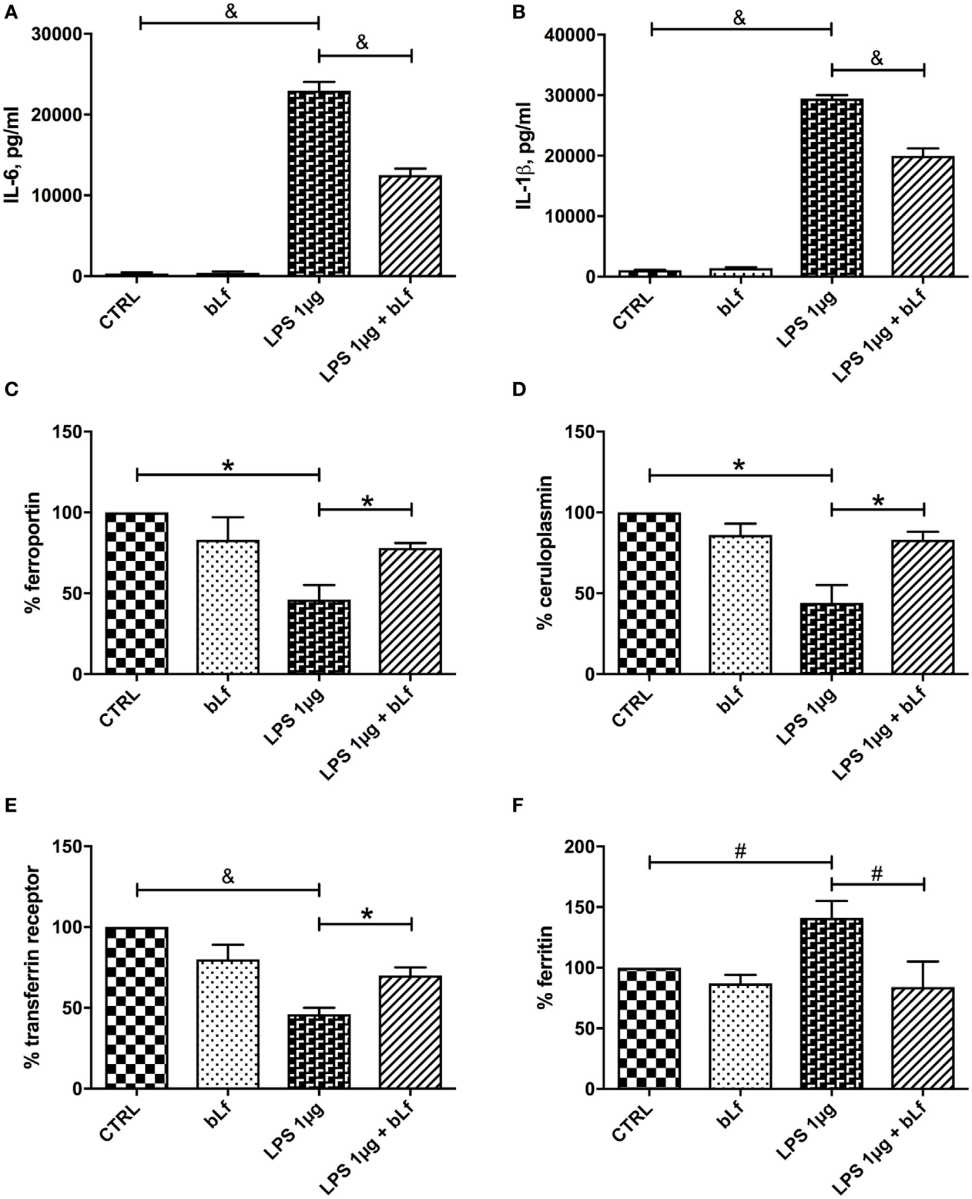
Changes in IL-6 **(A)**, IL-1β **(B)**, ferroportin **(C)**, membrane-bound ceruloplasmin **(D)**, transferrin receptor 1 **(E)**, and cytosolic ferritin **(F)** levels in THP-1 cells stimulated with LPS 1 μg/ml, in the presence or absence of 100 μg/ml bLf. ^#^*p* < 0.05; *p < 0.01; ^&^*p* < 0.001.

In order to assess whether the observed effects were confined to pro-inflammatory macrophages induced by the LPS/IFN-γ mixture, we treated differentiated THP-1 with high concentrations of LPS alone. Under these conditions, macrophages likely evolve into a heterogeneous population with phenotypes ranging from pro-inflammatory M1 to tolerogenic M2 (28). THP-1 cells were, therefore, stimulated with 1 µg/ml LPS, a concentration mimicking an infection from Gram-negative bacteria, in the presence or absence of 100 µg/ml bLf, and production of IL-6 and IL-1β as well as expression of Fpn, TfR1, Ftn, and Cp-GPI was assessed. As already observed with the IFN-γ/LPS mixture, a significant increase of IL-6, IL-1β, and Ftn and a parallel decrease of TfR1, Cp-GPI, and Fpn occurred upon LPS stimulation, all these effects being significantly prevented in the presence of bLf (Figure 4).

As with the stimulation with the LPS/IFN-γ mixture, no effect on IL-6, IL-1β, Fpn, TfR1, Ftn, and Cp-GPI production was detected in the control cells treated with bLf alone.

The changes of IL-10 observed upon stimulation with 1 µg/ml LPS were at odd with those observed with the LPS/IFN-γ mixture. As shown in Figure 3, while the inflammatory mixture grossly quenched IL-10 production (Figure 3A), LPS alone at 1 µg/ml concentration enhanced instead the cytokine synthesis more than 10-fold (Figure 3B). bLf had in this case the opposite effect, rebalancing the IL-10 levels toward lower values (yet much higher than in control, untreated cells).

**Figure 5:**
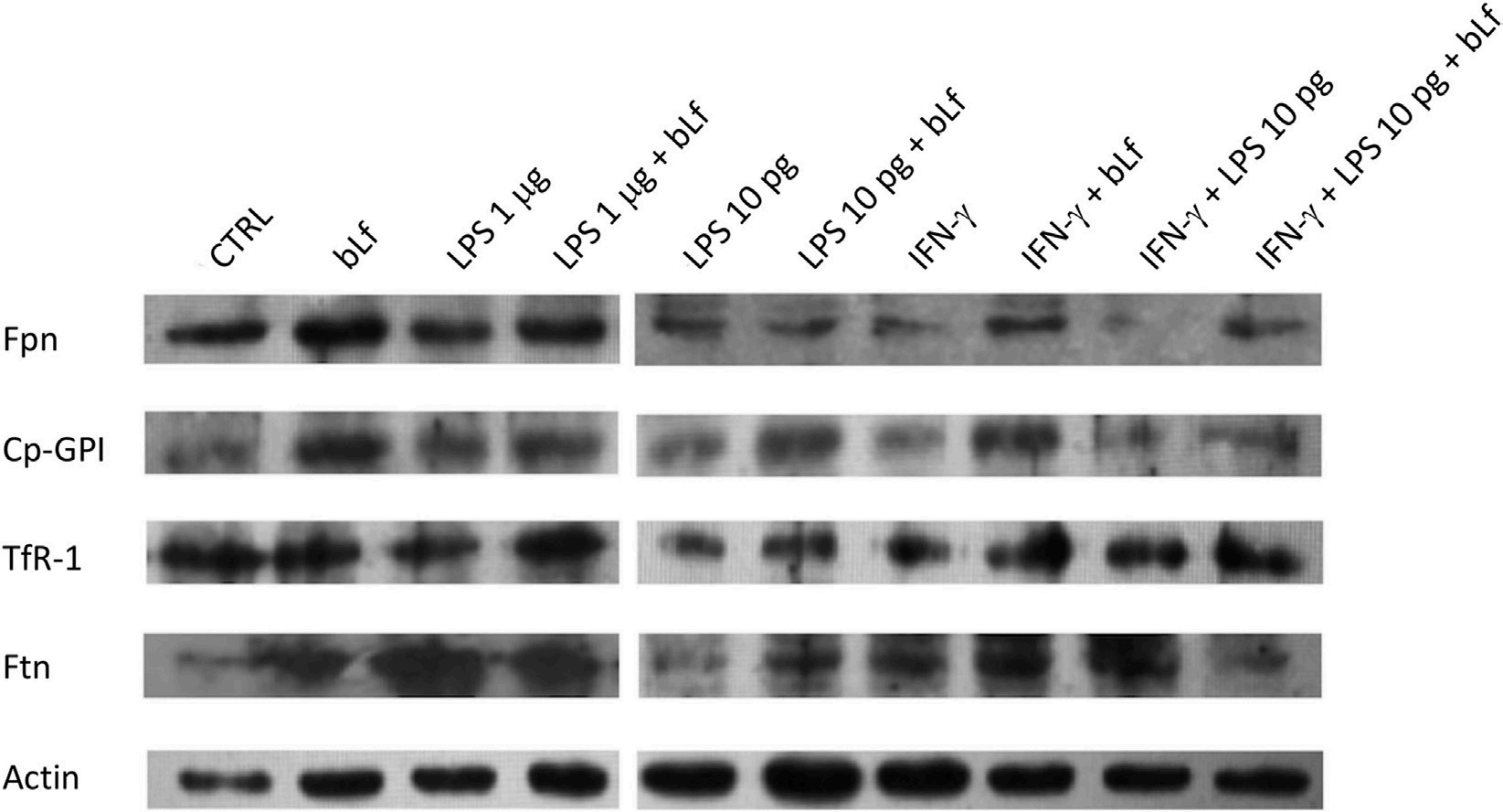
Representative Western Blot assays on ferroportin (Fpn), membrane-bound ceruloplasmin (Cp), transferrin receptor 1, and cytosolic ferritin (Ftn) in THP-1 cells stimulated both with a mixture of 20 ng/ml IFN-γ and 10 pg/ml LPS or with LPS 1 µg/ml, in the presence or absence of 100 µg/ml bovine Lf (bLf).

Representative Western Blot assays on Fpn, TfR1, Ftn, and Cp-GPI in THP-1 cells stimulated both with a mixture of 20 ng/ml IFN-γ and 10 pg/ml LPS or with LPS 1 µg/ml, in the presence or absence of 100 µg/ml bLf are shown in Figure 5.

## Discussion

Microorganisms require iron for the biosynthesis of macromolecules, thus pathogens have developed efficient systems to take up iron from the host. In turn, hosts rely on innate immune strategies that limit iron availability by finely regulating host genes involved in iron homeostasis. In this respect, it is of utmost importance to analyze how macrophages, which are crucial cellular types of the innate immune system, respond to inflammatory stimuli. Our data clearly show that a whole set of proteins involved in iron homeostasis is coordinately regulated upon stimulation of macrophages with inflammatory triggers. These changes take part into intracellular iron overload, a very unsafe condition *in vivo*, leading to higher host susceptibility to infections as well as to the IDA or AI.

Here, we clearly demonstrate that the anti-inflammatory effect of bovine lactoferrin is exerted on all examined components of the iron homeostasis machinery. It should be reminded that macrophages are a heterogeneous population of immune cells resulting from cytokine stimulation and pathogen or its products sensing. Indeed, depending on the microenvironment, macrophages are able to polarize into active subpopulations, with a continuum of macrophage subsets ranging from pro-inflammatory M1 to regulatory/anti-inflammatory M2 phenotypes. Stimulation of macrophages with low-dose LPS and IFN-γ leads to classical pro-inflammatory M1 polarization, while the widely used high-dose LPS stimulus gives rise to a more heterogeneous phenotype (28). It has been reported that, depending on M1 or M2 polarization, macrophages express at different extent a set of genes related to iron homeostasis leading to an “iron-retention” or an “iron-release” phenotype, respectively (29, 30). Thus, the cellular iron homeostasis machinery appears to be tightly regulated by inflammatory stimuli, with different outcomes depending on macrophage phenotype.

In this work, we demonstrate that bovine lactoferrin affects the expression of Fpn, TfR1, Ftn, and Cp-GPI in “pure” M1 macrophages obtained by stimulation with low-dose LPS and IFN-γ as well as in a more heterogeneous macrophage population, challenged with high-dose LPS.

In line with the expected “iron-retention” phenotype, levels of Ftn are increased while TfR1, Fpn, and Cp-GPI are decreased in M1 macrophages (low-dose LPS and IFN-γ). We demonstrate that bovine lactoferrin efficiently counteracts the effect of LPS and IFN-γ, restoring Fpn, Cp-GPI, Ftn, and TfR1 levels to those of unstimulated, uninflamed cells. The decrease of the anti-inflammatory cytokine IL-10 induced by low-dose LPS and IFN-γ is also counteracted by bovine lactoferrin.

Also in the case of stimulation with high-dose LPS, the phenotype appears to be one of “iron-retention” with higher levels of Ftn and lower expression of iron uptake/export proteins TfR1, Fpn, and Cp-GPI. Cytokines IL-6 and IL-1β are expressed at much higher levels compared to low-dose LPS/IFN-γ, indicating strong inflammatory conditions. Again, bovine lactoferrin dampens these effects, reducing the changes observed in its absence. In this case, however, levels of IL-10 significantly increase upon LPS stimulation [in line with other reports, e.g., Ref. (28)] and the presence of bovine lactoferrin only partially prevents the increase. This result suggests that, at variance with stimulation with the LPS/IFN-γ mix above, treatment with high doses of LPS triggers an endogenous anti-inflammatory response in THP-1 mediated by IL-10 and finalized to counteract the massive increase of IL-6 and IL-1β and of other pro-inflammatory factors.

It is interesting to note that bovine lactoferrin exerts its effect both under conditions mimicking the initial stages of infection (i.e., treatment with high doses of sole LPS) and when an inflammatory outcome has been set up (i.e., treatment with low doses of LPS and with IFN-γ). This suggests that, within this experimental framework, bovine lactoferrin acts as an anti-inflammatory agent able to both prevent the onset of inflammation and to relieve it once it has been established.

A last comment deserves to be made regarding the effect of inflammatory stimuli on Cp. Cp is well known to be a positive acute-phase reactant, i.e., a protein upregulated in the systemic response which usually follows a physiological condition that takes place in the beginning of an inflammatory process. Thus, Cp levels are greatly increased in plasma under inflammatory conditions (31, 32). On the other hand, we observe a decrease of the GPI-linked isoform in inflamed macrophages. This finding suggests that it is possible that the soluble and membrane isoforms of Cp are subject to differential regulation, and this could be related to the multifunctional nature of this multicopper oxidase. In plasma, it would play a major antioxidant role; therefore, high levels of Cp are required to scavenge reactive oxygen species produced in inflammation; in specialized cells such as macrophages, the predominant role of Cp-GPI would be linked to its ferroxidase activity in combination with Fpn to allow efficient iron release from cells to circulation and loading onto transferrin, thus avoiding intracellular iron overload as well as IDA and AI. Thus, it makes sense that in this latter case, bovine lactoferrin in inflamed macrophages restores the synthesis for both Cp-GPI and Fpn.

Overall, the capacity of bovine lactoferrin to reduce pro-inflammatory cytokine production and to prevent the changes of the whole set of proteins involved in iron homeostasis, in inflamed macrophages, underlines the pivotal role of this natural compound in the complex orchestration of iron and inflammatory homeostasis. Several drugs capable of modulating macrophagic phenotypes are emerging as attractive molecules for treating inflammation, and in this sense, bovine lactoferrin is no exception.

## Author Contributions

PV, MP, FB, and GM conceived and designed the experiments; AC, LR, ML, and MS performed the experiments and analyzed the data; PV and GM coordinated data collection and data quality assurance; AC, PV, and GM wrote the first draft of the manuscript. All authors read and approved the final version.

## Conflict of Interest Statement

The authors declare that the research was conducted in the absence of any commercial or financial relationships that could be construed as a potential conflict of interest.
